# Haloperidol is not the “one drug fits all” solution in the treatment of delirium

**DOI:** 10.1186/s13054-025-05413-8

**Published:** 2025-04-23

**Authors:** Johannes Ehler, Axel Petzold

**Affiliations:** 1 Department of Anesthesiology and Intensive Care Medicine, University Hospital Jena, Jena, Germany; 2 Department of Molecular Neuroscience, The National Hospital for Neurology and Neurosurgery, Queen Square Institute of Neurology, Moorfields Eye Hospital, UCL, London, UK

Dear Editor,

We read with great interest the recent publication by Cheng et al., investigating the role of haloperidol in delirium [1]. We acknowledge the authors’ diligent selection of studies for comparison, but wonder that landmark studies came to a diametrically opposed conclusion [2, 3]. There is need for a balanced interpretation of the presumed meta-analytical benefits of haloperidol regarding survival and delirium duration, with the interpretation of presumably fewer serious adverse events (SAEs) [1–3]. There are three key questions on which we would like to learn the authors’ opinion.

Firstly, the consistent comparison of haloperidol against placebo, while providing a baseline, overlooks the significant evolution towards a multimodal approach in delirium therapy. Current best practices emphasize a holistic strategy integrating non-pharmacological interventions for both prevention and treatment, with pharmacological agents reserved for specific indications [4]. The study does not address the contemporary core delirium care, particularly the extent to which non-pharmacological strategies were implemented. This omission is critical, given the increasing emphasis on non-pharmacological recommendations by leading societies such as the European Society of Anaesthesiology and Intensive Care (ESAIC) [4]. Does focusing solely on pharmacological intervention risks not present an incomplete and potentially biased perspective on delirium management?

Secondly, the observation of a reduced number of rescue benzodiazepine treatments through application of haloperidol warrants further scrutiny. Do benzodiazepines itself not have a pro-delirogenic potential and are therefore discouraged for treatment of delirium [4]?

Thirdly, the lack of a significant effect on duration of ventilation raises important questions, particularly concerning the broader context of ICU management. Prolonged ventilation is a multifactorial issue. Does attributing it solely to delirium not overlook the potential influence of sedative medication dosages, paradoxically including haloperidol itself?

Furthermore, we have concerns regarding the presentation of data in Table 2 on prevention of delirium [1]. The presentation of "benefits (%)" might be misleading when the Risk Ratio lacks significance. Similarly, the "any benefit" descriptor in Table 1 [1], while highlighting potential advantages, could be misinterpreted as a definitive recommendation for universal haloperidol use. In clinical practice there will be no “one drug fits all solution”.

In conclusion, we believe that while the study provides valuable data on the efficacy of haloperidol compared to placebo [1], a balanced interpretation should also include:

- Current guidelines increasingly support non-pharmacological strategies as the cornerstone of delirium management [4], moving away from a "one drug fits all" approach.
- Haloperidol carries a risk of QTc prolongation, necessitating careful consideration in high-risk populations, such as cardiac surgery patients who exhibit a high incidence of delirium. Neuroleptic malignant syndrome (NMS) is another severe complication.
- Haloperidol should be viewed as a potential component of a multimodal delirium management strategy and not advocated as a singular treatment solution.
- Future research should prioritize comparing multimodal delirium care with and without the addition of a specific drug like haloperidol among others, to better define its role within a comprehensive treatment framework.

We are looking forward to reading the authors’ thoughts on these points for a more balanced understanding of the role of haloperidol in contemporary delirium management and for guiding future research endeavors in this critical area.

## Data Availability

No datasets were generated or analysed during the current study.

## Abbreviations

ESAIC: European Society of Anaesthesiology and Intensive Care
NMS: Neuroleptic malignant syndrome
SAE: Serious adverse event
